# Supplementary material for: Optimization of High-Frequency Ultrasound Imaging to Detect Incremental Changes in Mineral Content at the Cartilage–Bone Interface Ex Vivo
Source: Biomimetics (Basel). 2025 Mar 5;10(3):160. doi: 10.3390/biomimetics10030160 (PMC11940126; doi:10.3390/biomimetics10030160)
Supplement: Supplementary file 1 [file biomimetics-10-00160-s001.zip › biomimetics-3417838-supplementary.pdf]

Table S1. Mean QUS parameters at baseline, re-scan and post-EDTA across different treatment conditions in adult bovine osteochondral explants (#p<0.05; ##p<0.01).

Treatment groups	Shoulder	Sample size (n)	Average Delta (mm)			Average CBI Intensity (dB)			Average Alpha (dB/mm)		
			Baseline	Rescan	Post-txt	Baseline	Rescan	Post-txt	Baseline	Rescan	Post-txt
**3h EDTA**	**Adult-3**	6	0.968	0.965	1.02 #	-10.90	-9.99	-9.34	177.4	179.7	195.5
7h EDTA	Adult-4	6	1.102	1.095	1.195 #	-10.71	-10.40	-10.89	188.6	183.3	187.5
3h PBS	Adult-3	3	0.969	0.979	0.976	-9.19	-9.34	-9.12	165.8	166.5	167.1
7h PBS	Adult-4	3	1.217	1.219	1.22	-10.38	-8.766	-8.893	187.3	189.7	184.1
2d EDTA	Adult-3	3	1.013	1.016	1.453 ##	-10.213	-10.187	-17.383	173.6	178.9	73.5 #
4d EDTA	Adult-4	3	1.266	1.27	1.693 ##	-11.893	-11.587	-18.373	179.5	180.1	59.9 #
2d PBS	Adult-3	3	1.05	1.063	1.08	-11.407	-10.913	-9.77	167.9	167.5	167.1
4d PBS	Adult-4	3	1.103	1.106	1.206	-10.393	-10.823	-11.133	174.0	171.9	179.2

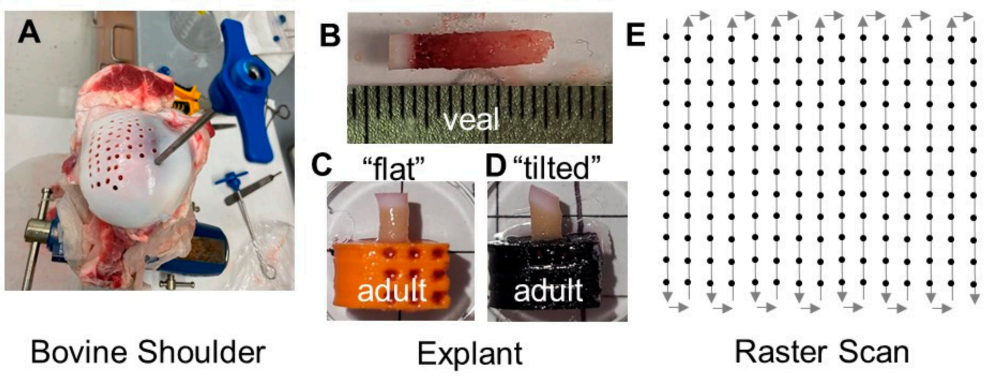

Figure S1. Macroscopic images of adult bovine humeral head explant extraction using (A) Jamshidi needle, example explants with (B,C) desired flat parallel cartilage surface or (D) unacceptable "tilted" surface and (E) example raster scan pattern. In this experiment, the raster scan covered a 6 × 6 mm area and used a 100 µm step size (the distance between the individual dots in panel E, where the arrows indicate the scan direction). Explants were press-fit in the 3D printed holder with a 3.2 mm hole in the center, ensuring that the CBI was at least 2 or 3 mm above the holder to prevent backscatter from the holder from interfering with the osteochondral images.

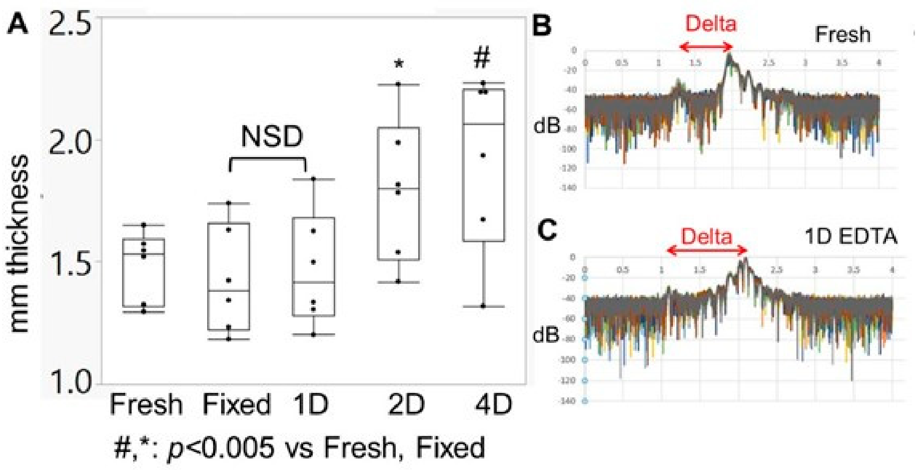

Figure S2. A pilot study using a 15 MHz transducer to measure HFUS adult bovine cartilage thickness failed to detect differences after 1 day of EDTA which decalcified the subchondral bone plate. (A) HFUS measures of cartilage thickness (QUS parameter Delta, mm, N=6) were generated from the distance between two major backscatter peaks of nine scan lines (B) before and (C) after EDTA incubation (N=6 adult bovine explants). HFUS cartilage thickness decreased by 60 ±195 µm after formalin fixation (p=0.60), increased slightly after 1 day of EDTA (42 ±46 µm vs. fixed explants, p=0.76), and increased significantly after 2 days of EDTA decalcification (369 ±56 vs fixed explants, p=0.001, N=6). NSD: not significant difference by matched pair test (N=6) after 1 day of EDTA which decalcified 263 µm of mineral below the tidemark. Graph shows the median (horizontal line), interquartile range (box), min-max (whiskers) and individual data points (dots). Abbreviations: * $p < 0.005$ two days (2D) EDTA *versus* fresh or fixed tissue before decalcification; # $p < 0.005$ four days (4D) EDTA *versus* fresh or fixed tissue; NSD: Non-significant difference after one day (1D) of EDTA decalcification; EDTA: ethylene diamine tetraacetic acid.

The wavelength of the sound pulse (λ) can be calculated using the formula:

$\lambda = c / f$ (1)

where

c=1600 m/s (we assume for cartilage) and (2)

f=15,000,000 (15 MHz), or f=35,000,000 Hz (35 MHz) (3)

Axial Resolution is calculated as follows:

$\lambda / 2$ (4)

Axial resolution for a 15 MHz transducer

λ = 1600 m/s / 15,000,000 Hz = 0.000106.7 m = 106.7 µm (5)

106.7 µm / 2 = 53.3 µm (6)

Axial resolution for a 35 MHz transducer

λ = 1600 m/s / 35,000,000 Hz = 0.000044 m = 45.7 µm (7)

45.7 µm / 2 = 22.85 µm (8)

Figure S3. Theoretical axial resolution of 15 MHz and 35 MHz transducers in cartilage, assuming a constant speed of sound at 1600 m/s.

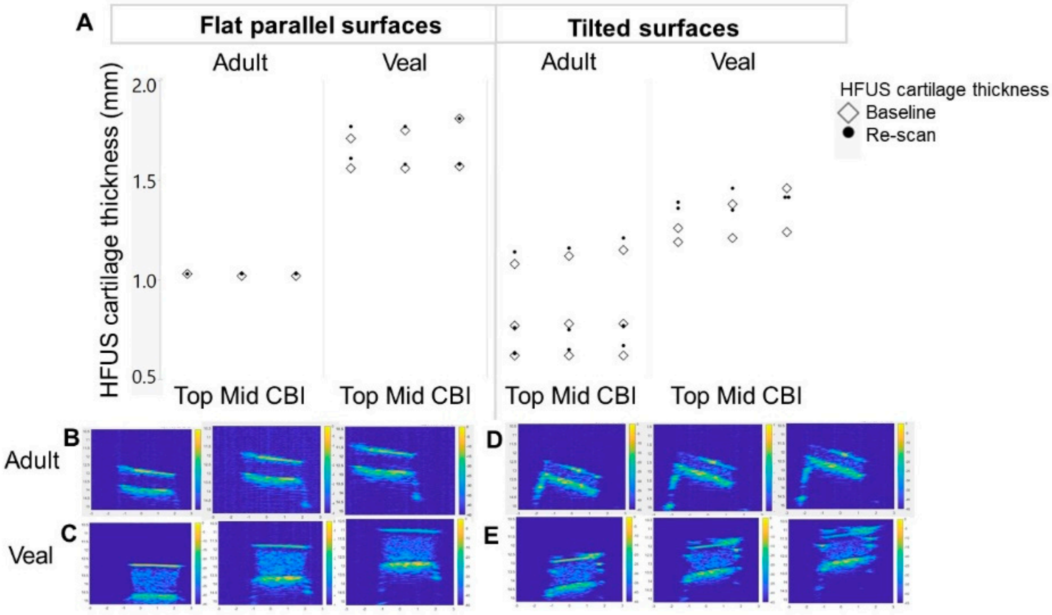

Figure S4. Evidence that HFUS cartilage thickness measures were highly reproducible at all 3 focal planes for explant samples with flat but not tilted surfaces. (A) Graph showing individual HFUS cartilage thickness measures (mm) for four veal and four adult explants stratified according to transverse explant images that showed an explant with (B,C) a flat surface or (D,E) a tilted surface. Abbreviations: Top (articular cartilage surface), Mid (mid-zone), CBI (cartilage-bone interface) level of focal depth for the 35 MHz transducer. Graph open diamond: HFUS cartilage thickness at baseline. Figure legend: Diamond: HFUS cartilage thickness of the baseline scan; Dot: HUFS cartilage thickness after the explant in the dedicated 3D printed holder was removed from the scan bath, reinserted into the complementary 3D-printed support, and re-scanned.

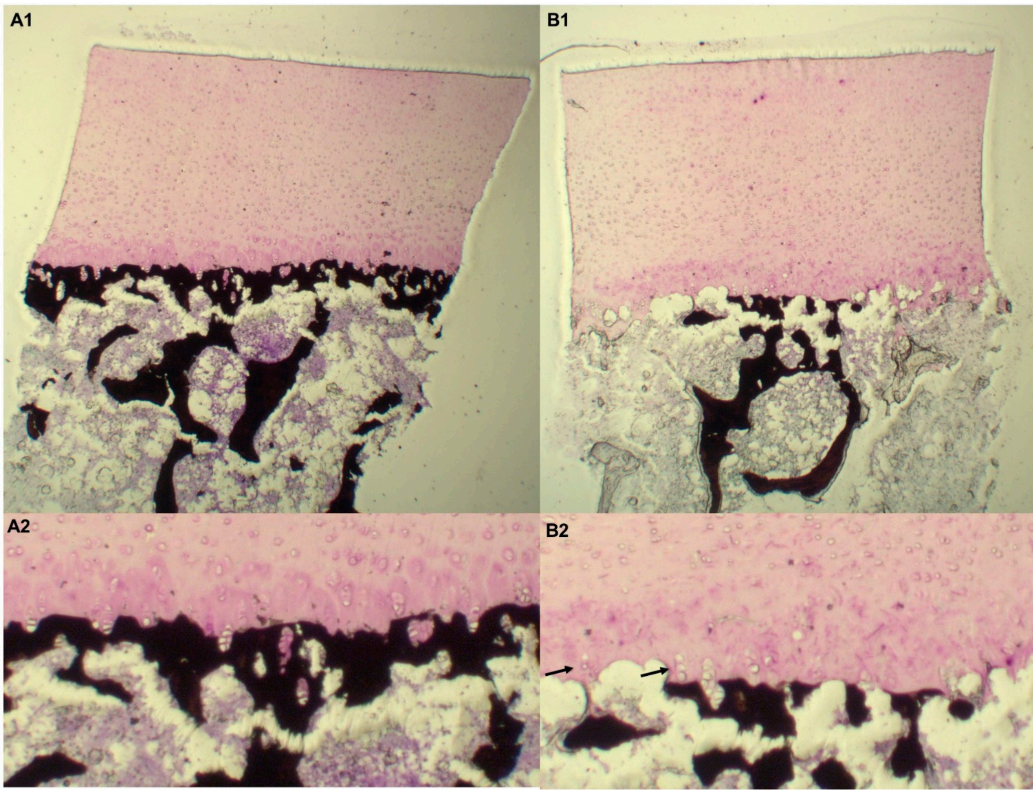

Figure S5. Enlarged histology images of von Kossa and DMMB-stained cryosections of the panels shown in Fig. 3D and 3H, showing incremental demineralization of the calcified cartilage layer after 4 h of EDTA treatment in the middle of a veal explant, but total decalcification at the explant edges. (A1,B1) 2.5x magnification images and (A2,B2) corresponding magnified images in the center of the explant, for no EDTA (A1,A2) and 4 h EDTA

(B1,B2). Arrows in panel (B2) indicate hypertrophic chondrocytes in the calcified cartilage layer that are no longer surrounded by mineral after 4 h EDTA exposure.

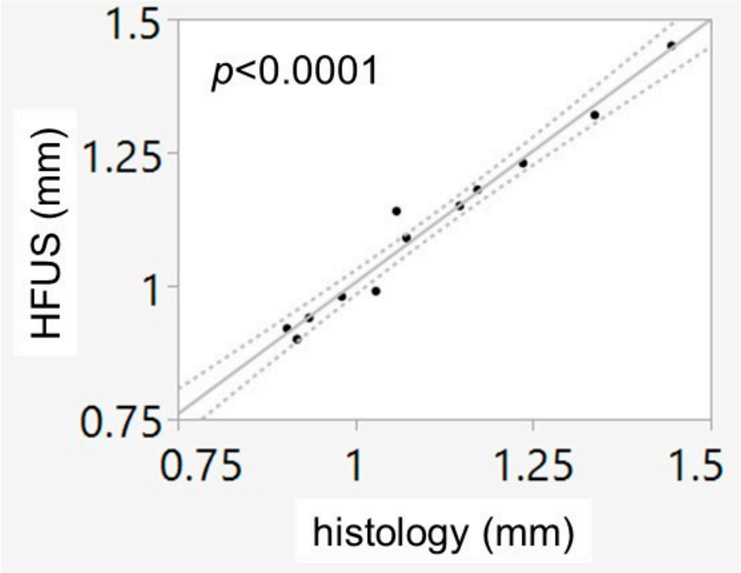

Figure S6. Graph showing that HFUS cartilage thickness after brief EDTA was positively correlated with non-mineralized cartilage thickness measured by histomorphometry. Pearson's correlation showed a relationship of HFUS thickness = 0.019 + 0.99 x histology thickness ($p<0.0001$, R = 0.98, $R^2 = 0.97$, N=12). Dotted lines: 95% confidence interval.

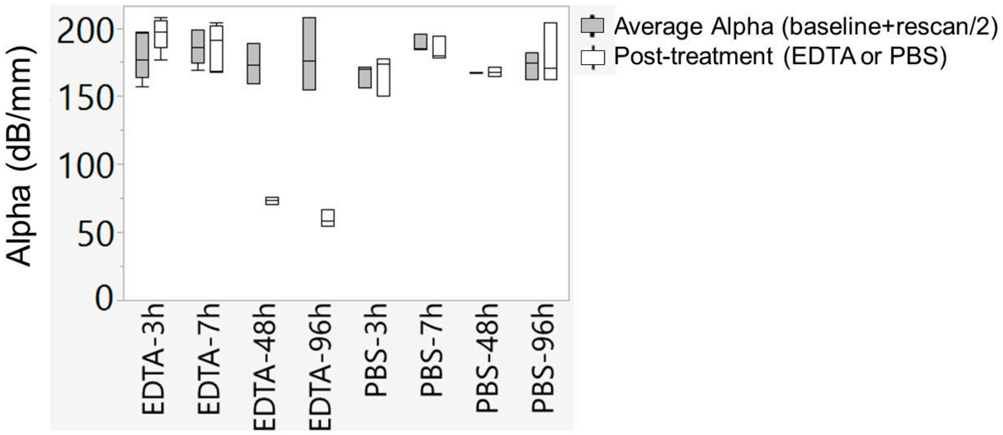

Figure S7. Alpha, the spatiotemporal increase in HFUS backscatter from 0.2 mm prior to the CBI and the CBI, was unchanged by brief EDTA treatment or PBS incubation, and significantly lower after full decalcification ($p<0.05$, matched pair test). Average HFUS Alpha measures after extended EDTA (n=3): 173.8 dB/mm (137.3 – 210.1 dB/mm, 95% C.I.) baseline vs. 73.5 dB/mm (66.8 – 80.1 dB/mm, 95% C.I.) post-48 h EDTA: 179.8 dB/mm (113.5 – 246.1 dB/mm, 95% C.I.) baseline vs. 59.9 dB/mm (43.8 – 75.9, 95% C.I.) post-96 h EDTA. The graph shows the median (horizontal line), inter-quartile range (box), and min-max (whiskers).

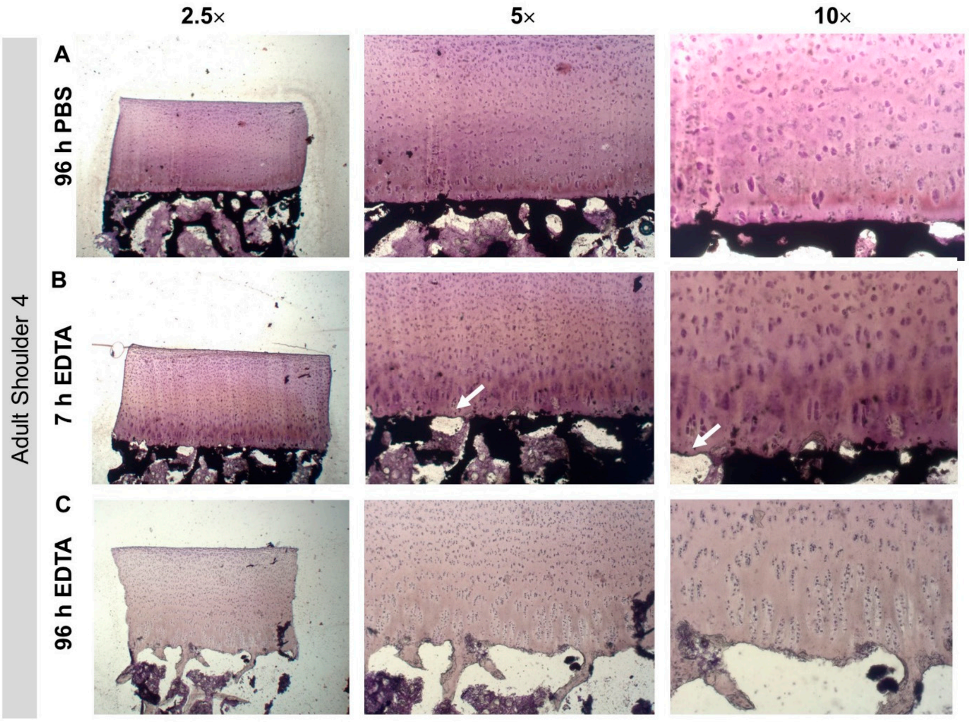

Figure S8. Histology images showing that brief surface-directed EDTA treatment of parafilm-wrapped explants led to incremental decalcification of the CBI without "outside-in" decalcification. Histological appearance of von Kossa (black mineral) and hematoxylin (purple) stained non-decalcified histology sections of 3 different explants from adult bovine shoulder 4, incubated for (A) 96 h in PBS, (B) 7 h in EDTA, or (C) 96 h in EDTA. White arrows in panels B (5 , 10) show a patch of CBI where the calcified cartilage was fully demineralized after 7 h of EDTA. These patches would be expected to show a shifted and reduced echogenicity in the *en face* segmented CBI images.

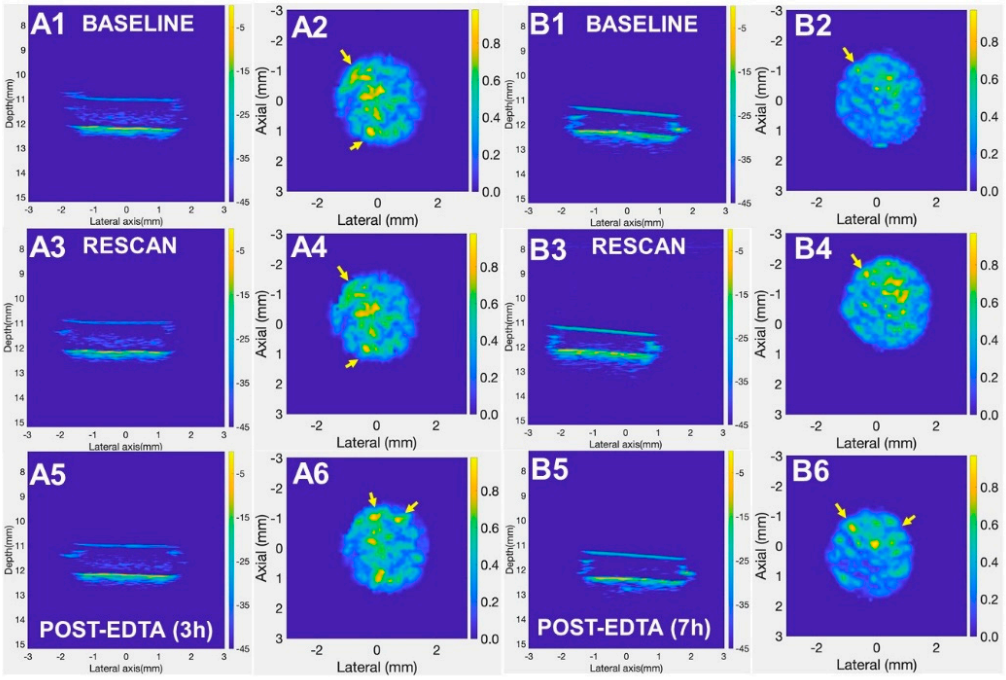

Figure S9. HFUS images showing that brief EDTA treatment of parafilm-wrapped bovine osteochondral explants resulted in a consistently altered CBI backscatter pattern across all samples (en face view), and "gap" in backscatter of the articular cartilage deep (radial) zone and demineralized calcified cartilage layer (transverse view). (A1,B1) show the baseline scan transverse HFUS images of adult bovine explants (color scale in dB) whereas (A2,B2) display the corresponding en face HFUS images at the CBI prior to 3 h or 7 h EDTA treatment (color scale in relative backscatter intensity in Volts, normalized to a scale from 0 to 1). (A3,B3) show the respective transverse HFUS images at re-scan, with (A4,B4) providing the matching en face views. (A5,B5) depict the transverse HFUS images post-EDTA treatment (3 h or 7 h, respectively), with (A6,B6) showing the en face HFUS images at the CBI post-EDTA treatment. Symbols: yellow arrows (A2,A4,A6) point to regions of high-intensity backscatter (yellow patches in the en face images) that have shifted after brief surface-directed decalcification (B2,B4,B6).
